# High-Grade Cervical Intraepithelial Neoplasia (CIN) Associates with Increased Proliferation and Attenuated Immune Signaling

**DOI:** 10.3390/ijms23010373

**Published:** 2021-12-29

**Authors:** Irene Tveiterås Øvestad, Birgit Engesæter, Mari Kyllesø Halle, Saleha Akbari, Beatrix Bicskei, Morten Lapin, Marie Austdal, Emiel A. M. Janssen, Camilla Krakstad, Melinda Lillesand, Marit Nordhus, Ane Cecilie Munk, Einar G. Gudlaugsson

**Affiliations:** 1 Department of Pathology, Stavanger University Hospital, 4011 Stavanger, Norway; saleha.akbari@sus.no (S.A.); bicskei.beatrix@hotmail.co.uk (B.B.); emilius.adrianus.maria.janssen@sus.no (E.A.M.J.); melinda.lillesand@sus.no (M.L.); marit.nordhus@sus.no (M.N.); einar.gudbjorn.gudlaugsson@sus.no (E.G.G.); 2 Section for Cervical Cancer Screening, Cancer Registry of Norway, 0304 Oslo, Norway; bieg@kreftregisteret.no; 3 Department of Obstetrics and Gynaecology, Haukeland University Hospital, 5053 Bergen, Norway; mari.halle@uib.no (M.K.H.); camilla.krakstad@uib.no (C.K.); 4 Centre for Cancer Biomarkers, Department of Clinical Science, University of Bergen, 5053 Bergen, Norway; 5 Department of Haematology and Oncology, Stavanger University Hospital, 4011 Stavanger, Norway; morten.lapin@sus.no; 6 Section of Biostatistics, Department of Research, Stavanger University Hospital, 4011 Stavanger, Norway; marie.austdal@sus.no; 7 Department of Chemistry, Bioscience and Environmental Technology, University of Stavanger, 4036 Stavanger, Norway; 8 Department of Gynaecology, Sørlandet Hospital, 4604 Kristiansand, Norway; Ane.Cecilie.Munk@sshf.no

**Keywords:** cervical cancer screening, CIN progression, differential gene expression, transcriptomic analysis

## Abstract

Implementation of high-risk human papilloma virus (HPV) screening and the increasing proportion of HPV vaccinated women in the screening program will reduce the percentage of HPV positive women with oncogenic potential. In search of more specific markers to identify women with high risk of cancer development, we used RNA sequencing to compare the transcriptomic immune-profile of 13 lesions with cervical intraepithelial neoplasia grade 3 (CIN3) or adenocarcinoma in situ (AIS) and 14 normal biopsies from women with detected HPV infections. In CIN3/AIS lesions as compared to normal tissue, 27 differential expressed genes were identified. Transcriptomic analysis revealed significantly higher expression of a number of genes related to proliferation, (*CDKN2A*, *MELK*, *CDK1*, *MKI67*, *CCNB2*, *BUB1*, *FOXM1*, *CDKN3)*, but significantly lower expression of genes related to a favorable immune response (*NCAM1*, *ARG1*, *CD160*, *IL18*, *CX3CL1*). Compared to the RNA sequencing results, good correlation was achieved with relative quantitative PCR analysis for *NCAM1* and *CDKN2A.* Quantification of NCAM1 positive cells with immunohistochemistry showed epithelial reduction of NCAM1 in CIN3/AIS lesions. In conclusion, *NCAM1* and *CDKN2A* are two promising candidates to distinguish whether women are at high risk of developing cervical cancer and in need of frequent follow-up.

## 1. Introduction

Nearly all cervical cancers are caused by a persistent infection with high-risk human papillomavirus (HPV) [1]. During their lifetime, the vast majority of sexually active women incur an HPV infection, but less than 10% develop persistent infection associated with a higher risk of developing cervical cancer [2]. Invasive cervical cancer is preceded by a long phase of pre-invasive disease named cervical intraepithelial neoplasia (CIN). CIN is sub-classed into CIN1, 2, or 3 reflecting increasing severity and is based on the proportion of abnormal cells populating the cervical epithelium. In Norway, there is a general increase in the prevalence of HPV, and a significant increase in the number of diagnosed CIN2/3 has been observed in the last 25 years [3]. CIN is classified as a pre-cancer, even though only approximately 9% of CIN1 will progress to CIN3 [4], and approximately 30% of CIN3 progress to cancer [5], while 20 to 40% of CIN3 will regress spontaneously [4,6,7]. Lesions with persistent HPV infection can progress into CIN3 and invasive cancer over a period of approximately 2–3 and 10–25 years, respectively [1,2]. This slow development, through several levels of cellular changes, makes cervical cancer a disease that could be marginalized with optimized HPV vaccination and screening [8]. 

Many countries are in the process of replacing primary cytology screening with a primary HPV-test. HPV-tests have increased sensitivity compared to cytology [9], and a negative HPV-test is a long-term predictor for not developing cervical cancer. HPV-tests have however, reduced specificity compared to cytology and to compensate, cytology is still used along with partial genotyping, as a triage test for HPV positive women [10,11,12]. Although the triage improves the risk stratification, a significant proportion of the women are incorrectly categorized as high-risk of developing premalignant lesions and cervical cancer and are exposed to unnecessary frequent follow-ups. In addition, an increasing number of HPV16/HPV18-vaccinated women will be entering the screening programs in the years to come. Altogether, the percentage of women with an HPV-infection with reduced oncogenic potential will rise, and the need for prognostic biomarkers for targeted identification of high-risk women is warranted.

The key to improved understanding of why some individuals harboring HPV infections develop high-grade CIN, while others provoke a successful immune response and clearance of the infection, is hidden in the complex interplay of the cells and molecules in the microenvironment of the lesion. To succeed in fulfilling its lifecycle and produce viral copies, HPVs have evolved different mechanisms to evade detection by host immune responses. First, to avoid detection by immune cells during replication, viral DNA and proteins are hidden in the host cell nucleus. Also, HPV oncogenes E5, E6 and E7 are important contributors for evasion of both host innate immunity, including dendritic (DC), Langerhans (LC) and natural killer (NK) cells, and downregulation of adapted immunity with decreased numbers of both T-helper cells (CD4+) and specific cytotoxic T-cells (CD8+), crucial for the elimination of HPV infected keratinocytes [13,14,15]. Furthermore, the pre-malignant cells recruit and remodulate non-malignant cells like fibroblasts and tumor infiltrating lymphocytes (TILs), promoting vascularization, extracellular matrix remodeling and development of pre-malignant lesions [16,17]. 

The objective of the present study was to investigate the expression of RNA transcripts related to the immune response in normal and high-grade cervical biopsies. The aim was to explore the mechanisms involved in the microenvironment of persistent HPV infections to better differentiate between women with high and low risk of developing high grade CIN and cervical cancer.

## 2. Results

### 2.1. HPV Positivity, Genotype and Cytological Diagnoses in the Study Cohort

**Figure 1:**
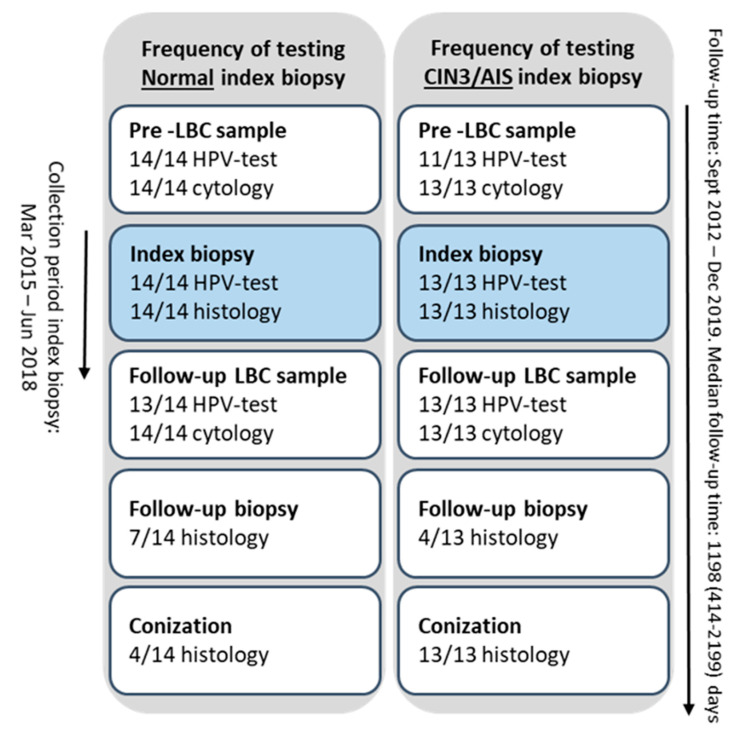
Overview of the index biopsies and additional cervical samples, before and after the index biopsies, from the women included in the study.

Twenty-seven biopsies, 14 normal, 12 CIN3 and one Adenocarcinoma in situ (AIS) case, were collected from the biobank “General biobank for cervical cancer and high grade Cervical Intraepithelial Neoplasia” (Figure 1) and are referred to as index biopsies. Additional cervical sample results from the corresponding women were collected from their medical records. 

Women with CIN3/AIS biopsies had more severe cytology diagnoses, including both high-grade squamous intraepithelial lesion (HSIL) and atypical squamous cells cannot exclude HSIL (ASC-H) and were more frequently infected with the most oncogenic HPV genotypes (HPV16 or HPV18) as compared to women with normal biopsies (Table 1). A similar trend for the genotypes was observed in the index biopsies, but was not significant. 

Furthermore, women with CIN3/AIS lesions were all treated, resulting in a significantly shorter time of HPV persistency, and all over they had a shorter follow-up time from the first HPV positive and/or abnormal liquid based cytology (LBC) to the last follow-up sample (Table 1). No statistical difference in age was detected between women with normal and CIN3/AIS biopsies (Table 1). 

HPV testing of DNA isolated from the normal biopsies, resulted in five HPV negative, seven HPV16 positive and two HPV52 positive samples (Table 1). Three biopsies were concordant with HPV16 positivity in both the LBC and in the biopsy, while two women were possibly concordant with non HPV16/18 positivity in the LBC and HPV52 in the biopsy. All the CIN3/AIS biopsies showed corresponding genotypes as detected in the LBC samples, apart from two samples diagnosed with HSIL without HPV testing. 

### 2.2. Gene Expression Using Oncomine Immune Response Research Assay

**Figure 2:**
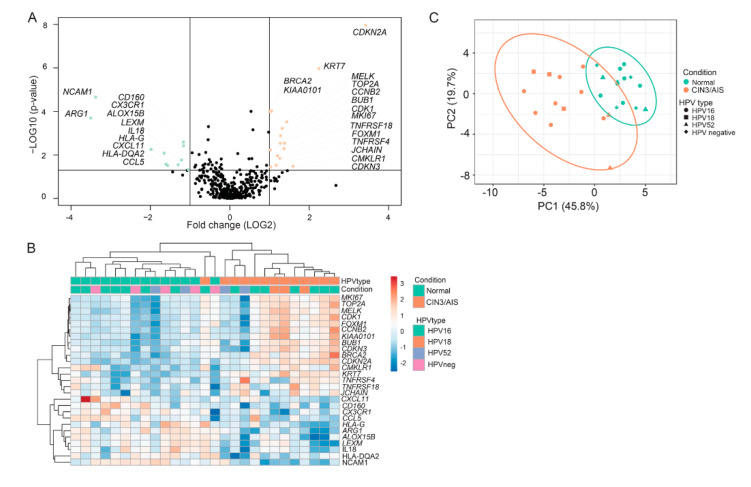
Gene expression from the 27 cervical biopsies (14 normal and 13 CIN3/AIS biopsies) were analyzed using Oncomine™ Immune Response Research Assay. (**A**) Distribution of *p*-values (−log10) as a function of fold change (log2) between CIN3/AIS and normal biopsies. Differentially expressed genes (DEGs, fold change > |2| and *p* < 0.05) showing downregulation in CIN3/AIS are depicted in green, whereas genes upregulated in CIN3/AIS are depicted in orange. (**B**) Hierarchical clustering based on the 27 significant DEGs with correlation clustering distance and average linkage. The HPV status is included; HPV16 (green), HPV18 (orange), HPV52 (purple) and HPV negative (pink). (**C**) Principal component analysis (PCA) of the first two principal components of the gene expression data. Each point represents a biopsy, normal (green), and CIN3/AIS biopsies (orange).

All 27 biopsies were subjected to RNA sequencing with the targeted amplicon based Oncomine™ Immune Response Research Assay [18], analyzing gene expression from 398 genes. Fold change in gene expression between normal and CIN3/AIS biopsies, together with *p*- and false discovery rate *(FDR)* adjusted *p*-values, were calculated by ANOVA using the TAC 4.0.2. Twenty-seven genes matched the criteria of fold change >|2| and *p* < 0.05 and were defined as differentially expressed genes (DEGs) (Figure 2A). To validate the differential gene expression, qPCR was performed for two selected genes (*CDKN2A* and *NCAM1*), and the normalized C_q_ method (2^−^^∆^^Cq^) was used to calculate the relative gene expression in normal and CIN3/AIS biopsies [19]. Spearman’s rho correlation between the RNA sequencing data and the qPCR results, showed good correlation for both selected genes (*CDKN2A*, r = 0.84, *p* < 0.001 and *NCAM1*, r = 0.86, *p* < 0.001). 

Of the 27 DEGs, 16 genes were upregulated, while 11 genes were downregulated in CIN3/AIS lesions compared to normal cervical tissue (Table 2 and Figure 2A). Unsupervised hierarchical clustering of the 27 DEGs yielded a heatmap with two main clusters (Figure 2B). Cluster 1 contains all normal lesions in addition to one CIN3 lesion. Cluster 2 contains only CIN3/AIS lesions. No correlation to HPV genotype were detected within the clusters. Principal Component Analysis (PCA) of the 27 DEGs also revealed two distinct clusters according to the conditions, persistent CIN3/AIS and normal tissue (Figure 2C).

The tumor markers *CDKN2A* and *KRT7* were the most upregulated genes with fold changes 10.7 and 4.8, respectively, while markers for proliferation (*MELK*, *CDK1*, *MKI67*, *CCNB2*, *BUB1*, *FOXM1*, *CDKN3*) had fold changes spanning from 2.0–2.7 (Table 2). Gene set enrichment analysis (GSEA) also revealed an enrichment of genes associated with cell cycle processes and proliferation in persistent CIN3/AIS (Table 3). All genes with downregulated expression in persistent CIN3/AIS were related to a favorable immune response (Table 2). The commonly used marker for natural killer (NK) cells, *NCAM1*, and the macrophage marker, *ARG1*, had the lowest fold changes of −10.4 and −11.3, respectively (Table 2).

### 2.3. IHC Analysis

**Figure 3:**
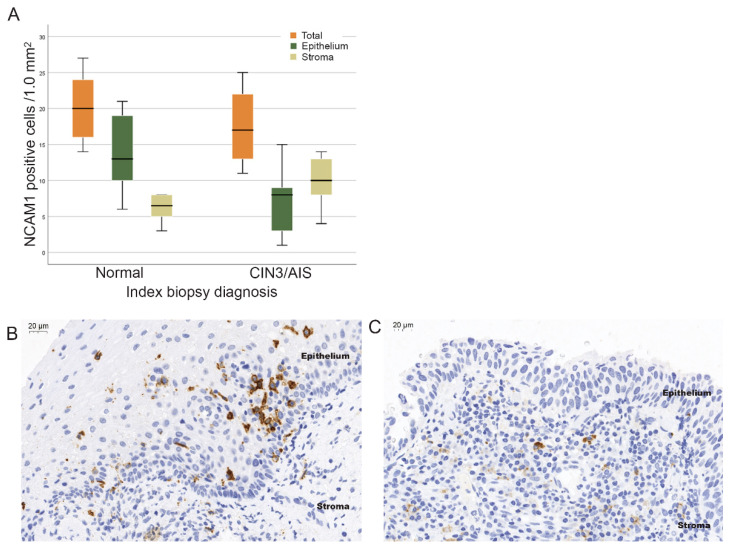
Protein expression of NCAM1. (**A**) Boxplots illustrating the distribution of NCAM1 positive cells/1.0 mm^2^ of normal and CIN3/AIS biopsies: in total (orange), in the epithelium (green) and in the stroma (olive green). Immunohistochemical staining of NCAM1 positive lymphocytes in (**B**) a normal biopsy and (**C**) a CIN3 biopsy (40× magnification).

NCAM1 was the most downregulated gene in CIN3/AIS lesions and to validate the observed reduction of NCAM1 mRNA in CIN3 lesions on the protein level, quantification of positive NCAM1 cells with IHC analysis was performed. The mean total number of NCAM1 positive cells in normal biopsies were 20/1.0 mm^2^ (range, 14–27), and in CIN3/AIS lesions, 17/1.0 mm^2^ (range, 11–25), showing no significant differences between the groups (*p* = 0.16) (Figure 3A). There were, however, a significantly higher number of positive cells in the epithelium of normal biopsies (14/1.0 mm^2^ (range, 6–21) vs. the epithelium of CIN3/AIS biopsies (8/1.0 mm^2^ (range, 1–15), *p* = 0.003). There were also a significantly higher number of NCAM1 positive cells in the stroma of CIN3/AIS biopsies (10/1.0 mm^2^ (range, 4–14) compared to normal biopsies (6/1.0 mm^2^ (range, 3–8), *p* = 0.003). The number of positive cells with strong expression of NCAM1 was also significantly higher in the epithelium of normal as compared to CIN3/AIS biopsies (11 strong and 3 weak vs. 1 strong and 12 weak, *p* < 0.001), but not in the stroma (*p* = 0.068). 

### 2.4. Ingenuity Pathway Analysis

Ingenuity Pathway Analysis (IPA^®^, Qiagen, Redwood City, CA, USA) was performed to test relationships between up- and downregulated genes and pathways relevant for the CIN3 phenotype relative to normal lesions. IPA was performed by selecting the feature “the relationship between cellular infiltration and proliferation of epithelial cells”.

**Figure 4:**
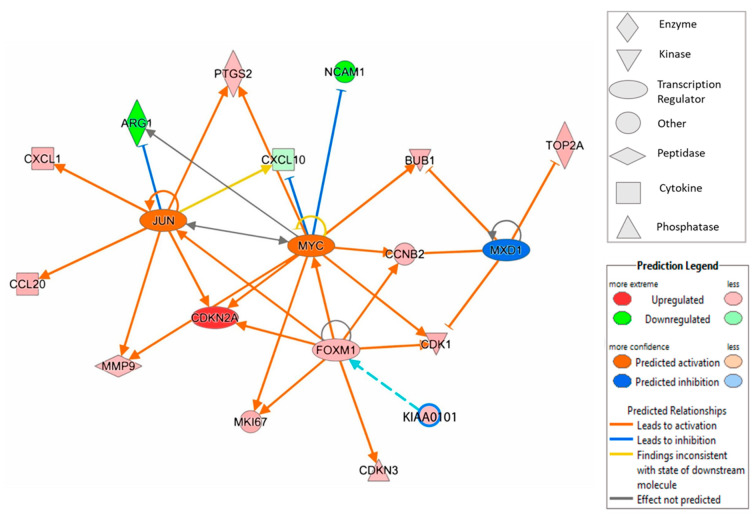
Ingenuity Pathway analysis (IPA) illustrates the association between the transcription factors FOXM1, JUN, MXD1 and MYC and differentially expressed genes in CIN3/AIS and normal biopsies, limited to z score, ≥2 (activation) and ≤−2 (inhibition) and overlay of KIAA0101.

The predicted model indicates modulation of the Myc-Max-MXD1 network of transcription factors. Myc was predicted to be activated, while the Myc agonist MXD1 was predicted to be inhibited (Figure 4). Altogether, the model predicts stronger regulations of genes modulated by Myc. 

Two additional transcription factors, FOXM1 and JUN, play important roles in the model (Figure 4). *FOXM1* shows a 2.3-fold upregulated expression in CIN3/AIS lesions (Table 2) and was predicted to activate Myc among others. FOXM1 is also modeled to regulate JUN, an activator of several molecules and inhibitor of ARG1, the most downregulated mRNA in CIN3/AIS lesions (Table 2).

## 3. Discussion

Unnecessary diagnostic biopsies, and sometimes even cone excisions, during follow-up of persistent HPV-infections and normal biopsies can be stressful for the affected women and are time consuming for both gynecologists and pathologists. An improved toolbox for better identification of women with increased risk for progression and cervical cancer development is warranted. The main objective for the present study was to compare the expression of key genes associated with immune control between normal and CIN3/AIS biopsies, but also including genes related to proliferation, cell cycle checkpoints, and cytokine signaling. A deeper understanding of the mechanisms involved in persistent HPV infections and the development of cervical high-grade lesions will strengthen the chances of identifying biomarkers with the ability to stratify between persistent HPV infections with and without the potential for developing into progressive high-grade lesions. 

In total, 27 genes were differentially expressed between normal and CIN3/AIS biopsies; 16 genes were upregulated, while 11 genes were downregulated in CIN3/AIS lesions. The Oncomine™ results were verified by qPCR for two genes (*CDKN2A* and *NCAM1*), and the differential expression of *NCAM1* was confirmed at the protein level, strengthening the validity of our results. Overall, our Oncomine™ results show that CIN3/AIS lesions as compared to normal cervical tissue, had higher expression of genes related to proliferation and tumor markers and lower expression of genes related to a favorable immune response, including T-cell activation, regulation and differentiation of immune cell infiltration. GSEA also identified increased proliferation in the CIN3/AIS lesions. 

The hierarchical clustering and PCA results show good separation in gene expression between normal and CIN3 biopsies. In the hierarchical clustering, normal and CIN3/AIS separate in two clusters, except for one CIN3 biopsy, clustering together with normal biopsies. A sub-cluster of three CIN3/AIS lesions resembles normal biopsies in gene expression related to proliferation, while the expression of genes related to immune response were in line with CIN3/AIS biopsies. 

NK cells play a key role as first line host defense against virus infected cells [20] and are involved in the expansion of T- and B-lymphocytes important for immune surveillance of HPV infections [21]. Interestingly, the Oncomine results showed down-regulation of several immune-related genes in CIN3/AIS lesions compared to normal lesions. For instance, *NCAM1* was more than 10-fold down regulated in CIN3/AIS lesions. Besides being a marker for NK cells, *NCAM1* is expressed by various T cells and dendritic cells (DCs), both important for immune surveillance of HPV infections [22]. Our IHC results showed, however, no differences in the total number of positive NCAM1 cells between normal and CIN3/AIS, but within the epithelium of normal biopsies, a significantly higher number of positive cells with strong expression of NCAM1 was found. This could explain the overall down-regulated gene expression of *NCAM1* in CIN3/AIS biopsies. Lima et al. identified two distinct phenotypes of NCAM1 positive NK-cells, NCAM1^hi^ versus NCAM1^lo^, and related NCAM1^hi^ to a subset of activated circulating NK-cells [23]. This is in agreement with Reiners et al. connecting reduced cytotoxicity of NK cells to downregulated expression of NCAM1 [24]. Increased numbers of circulating NCAM1 negative NK cells have also been found in patients with various virus infections and among elderly, but not in healthy individuals [25,26,27,28]. Altogether, these studies emphasize the association between *NCAM1* expression and the effector function of NK cells and support decreased activity of NK cells in CIN3/AIS lesions in our study. 

This assumption was further strengthened with decreased expression of *CX3CL1* in the CIN3/AIS biopsies. *CX3CL1* is expressed on mature DCs and by promoting activation of NK cells, plays a crucial role in the NK/DCs interaction [29,30]. In addition, *CD160*, an NK receptor, was also downregulated in the CIN3/AIS lesions. *CD160* together with *IL18*, also downregulated in the CIN3/AIS lesions, are both essential for NK cell mediated IFN-γ production [31,32]. The production of IFN-γ by activated NK cells promotes a cytotoxic T-cell response and the development of memory T-cells involved in the adapted immunity [21]. In summary, our results suggest that in CIN3/AIS lesions the NK cells are less effective, lacking the ability to migrate into the epithelium [22] which in turn hampers their capability to activate T-cells. 

Weakened immune response in CIN3/AIS lesions is further supported by the strong downregulation of *ARG1* in the CIN3/AIS biopsies. *ARG1* is expressed by macrophages, and downregulates immunosuppressive cytokine production (e.g., IL-4, IL-5, and IL-13) by T-helper cell 2 (Th2) in response to infectious disease [33]. Reduced *ARG1* in CIN3/AIS lesions is likely a sign of chronic inflammation and downregulated immune response, promoted by Th2 cell types caused by persistent and unresolved HPV infections [34]. Enhanced weakening of the adaptive immune system is also suggested by decreased expression of key inflammatory chemokines CCL5 and CXCL11 in the CIN3/AIS lesions. Together with CXCL10 and CXCL9, they are essential for the recruitment of effector CD4+T helper cells, CD8+ cytotoxic T-cells and NK cells [35,36]. Altogether, our results imply that the CIN3/AIS lesions tend to have an immune-suppressive environment.

The tumor marker *CDKN2A* had the highest fold change in CIN3/AIS as compared to normal biopsies. This gene encodes the tumor suppressor p16INK4a, a cyclin-dependent kinase inhibitor regulating G1/S transition. p16INK4a becomes overexpressed as a result of inactivation and degradation of the cell-cycle regulatory retinoblastoma protein (pRb) by the HPV *E7* oncogene [37]. The transcription factor group E2F, normally bound and inactivated by Rb, can bind the promoter region facilitating the increased expression of p16INK4a. Another gene with significantly upregulated expression in CIN3/AIS lesions was *MKI67*, a marker for cell proliferation. MKI67, together with p16INK4a, are commonly used as immunohistochemical markers in CIN diagnostics, and are associated with the grade of dysplastic changes in the epithelium [6,7], but their ability to predict the outcome of an HPV infection or a CIN lesion is limited [6]. Interpretation of immunohistochemical biomarkers is subjective and in need of fine tuning to reach acceptable sensitivity and specificity levels [38,39]. 

Other genes identified in this study as over-expressed in CIN3/AIS lesions compared to normal cervical tissue are also associated with tumor development (*KRT7*, *TNFRSF18* and *TNFRSF4)*. The tumor marker *KRT7* is found to be upregulated in a small population of squamous columnar (SC) junction cells, as compared to other squamous and columnar cells of the transformation zone. SC junction cells are described to have a unique expression profile with embryonic characteristics involved in cervical carcinogenesis [40]. TNFRSF18 and TNFRSF4 belong to the tumor necrosis factor receptor super family [41]. They are both expressed on T regulative lymphocytes (Tregs), an immunosuppressive subset of T cells, and promote chronic inflammation associated with HPV persistence, cancer development and antitumor effects through suppression of effector cytotoxic T cells (CTL) [42]. 

Zhao et al. identified TOP2A, CDK1, BUB1 and CCNB2 as candidate biomarkers for cervical cancer progression in a study using the Gene ontology and Omnibus database [43]. All four genes showed significantly higher expression in CIN3 as compared to normal biopsies in the current study. Specifically, TOP2A, a promoter of epithelial-mesenchymal transition (EMT), has been associated with increased invasive properties of cancer cells, and has been suggested as a prognostic factor for cervical cancer [44]. Furthermore, *TOP2A* combined with *CDKN2A* mRNA expression showed high sensitivity and specificity for HSIL [45] in LBC samples. 

*KIAA0101* and *FOXM1* are both known for their oncogenic properties. The transcriptional factor FOXM1as a driver for angiogenesis, invasion and metastasis [46], associated with poor prognosis in early-stage cervical cancer. KIAA0101, a promoter of microvascular invasion by inducing EMT and a critical target of FOXM1 [47], is involved in cell proliferation, cell survival, DNA repair and function as a potential oncogene. 

The IPA diagram presented in this study, illustrates how FOXM1 and the proto-oncogene AP-1 transcription factor subunit, Jun, in addition to the Myc/Max/MXD1 network of transcription factors, play important roles in for the expression of DEGs in the current study. The proto-oncogene transcriptional factor Myc activates genes related to proliferation such as *CDKN2A*, *CDK1*, *MKI67*, *MMP9*, *BUB1* and *CCNB2*, while the expression of non-inflammatory genes *NCAM1* and *CXCL10*, are inhibited. By antagonizing the Myc mediated transcriptional activation, MXD1 (Mad1) acts as a modulator by inhibiting activation of genes related to proliferation and transformation, such as *TOP2A*, *BUB1* and *CCNB2* [48], all of which were found to be upregulated in CIN3/AIS in this study. Furthermore, Jun acts as a fine tuner of macrophage activation by inhibiting ARG1 [49], which was found to be upregulated in the normal biopsies. 

Collectively, the differentially expressed genes in the CIN3 biopsies, suggest an environment with increased proliferation, increased plasticity, invasive properties and reduced immunological activity. 

A limitation of the study is the technique (macro dissection) used for the collection of tissue for RNA isolation. The density and number of cell types in the epithelial and stromal compartments differ in the isolated mRNA. Normal biopsies have a limited number of proliferating cells in the epithelium and CD34+fibrocytes are widely distributed in the stroma [50]. The CIN3/AIS biopsies, however, have high numbers of proliferating cells in the epithelium and many different immune cells can be observed in the stromal compartment, while a decreasing density of CD34+ fibrocytes has been observed [6,7,50]. In addition, the mRNA isolated from the epithelium and stroma is pooled. Furthermore, Oncomine only includes a subset of 398 immune related genes, thus other transcriptomic differences between normal and CIN3/AIS biopsies will not be revealed. Lastly, the moderate number of observations included hampers the strength of the results. A higher number of samples would for instance increase the chance of detecting the potential impact of HPV genotypes. 

Strengths of the current study include that the two compared cohorts were distinct. Women with normal biopsies remained normal/CIN1 throughout the observation period. The CIN3/AIS biopsies were from otherwise healthy women; with a first-time onset, histologically (p16/Ki67 supported) confirmed CIN3/AIS, evaluated by an experienced pathologist. The CIN3/AIS diagnoses were all confirmed in the cone excisions. Furthermore, the unique characteristics of this study, make it highly relevant as a starting point for the detection of prognostic tools for the development of high grade CIN. 

We have identified differentially expressed RNA transcripts related to proliferation and immune responses in HPV positive women with normal or CIN3/AIS biopsies. The DEGs are all potential prognostic biomarkers for high-grade CIN and cervical cancer development. *NCAM1* together with *CDKN2A* are the most promising candidates given the highest fold changes between the groups. However, they need to be validated retrospectively in larger independent cohorts of HPV positive women with normal or CIN3 cervical lesions using techniques already established in clinical laboratories, such as IHC and qPCR. The development of robust prognostic biomarkers would not only benefit a large number of women by providing better cervical cancer prevention, but also reduce the burden on hospital staff and enable more efficient budgeting.

## 4. Material and Methods

### 4.1. Biological Material

From March 2015 until June 2018, formalin-fixed, formalin-fixed, paraffin-embedded (FFPE) biopsies from a cohort of 355 women attending the Norwegian cervical cancer screening program (NCCSP), were prospectively collected at the Gynecologic outpatient clinic, Stavanger University Hospital (SUH), Stavanger, Norway, and stored in the biobank “General biobank for cervical cancer and high-grade Cervical Intraepithelial Neoplasia” (2016/805/REC). Written informed consent was received by inclusion. Twenty-seven women were selected from the biobank to the current study based on their biopsy diagnosis and screening history. Fourteen women had biopsies scored as normal, 12 had CIN3 and one had an AIS biopsy (Figure 1). 

The follow-up screening history during a median observational period of 1198 (414–2199) days were retrieved from the laboratory data system at the Pathology Department at SUH (Unilab 700). The screening history included the number and results of (1) cytology samples, Atypical Squamous Cells of Undetermined Significance (ASC-US), Low-grade intraepithelial lesion (LSIL), ASC-H, HSIL and atypical glandular cells of undetermined significance (AGUS), (2) HPV tests (HPV negative, HPV16 or 18 positive, non 16/18 HPV positive), (3) diagnostic biopsies (normal, CIN1, CIN3 and AIS and 4) cone excisions. 

Follow-up time was defined as the time period between the first HPV positive and/or abnormal LBC, retrieved from the laboratory data system, and the last follow-up sample. The reported observation period for the included women is from September 2012 until December 2019. The study is approved by the Norwegian Regional Ethics Committee, Rec West, (2016/805/REC); (2019/264/REC) and (2019/10399/REC). 

### 4.2. RNA/DNA Isolation

The “Recover all total nucleic acid isolation” kit (Invitrogen, Thermo Fisher Scientific, Waltham, MA USA) was used for simultaneous RNA and DNA isolation. Isolation was performed according to the manufacturer‘s protocol. For CIN3/AIS biopsies, nucleic acids were isolated from (5–10) × (4–5 µm) µm sections from the FFPE tissue block, macro dissected from the most severe dysplastic area of the epithelium and the adjacent stroma. For normal biopsies, nucleic acids were isolated from (2–5) × (4–5 µm) sections comprising all the biopsies in the FFPE tissue block. To quality assure the normal and CIN3/AIS diagnoses, adjacent sections to the sections used for isolation, were Hematoxylin/Eosin (HE) stained and examined by the pathologist. 

### 4.3. HPV Testing

DNA isolated from the biopsies was used for genotyping, using the InnoLipa HPV detection system (Fujirebio Europe N.V., Gent, Belgium). InnoLipa is an automated line probe assay, based on the reverse hybridization principle, for the detection of 32 different genotypes including high-and low-risk and probably high-risk HPV. HPV testing of ThinPrep LBC follow-up samples, was performed on the cobas 4800 fully automated system (Roche Molecular Diagnostics, Pleasanton, CA, USA) was used for HPV testing. The cobas 4800 simultaneously detects 14 HPV genotypes, including specific identification of HPV16 and HPV18. 

### 4.4. Functional RNA Quantification

RNA quantification assays were used to quantify the exact amplifiable RNA concentrations from the FFPE samples. A one-step real time quantitative PCR (RT-qPCR) procedure was applied (LightCycler^®^ 480 System, Roche Diagnostics, Rotkreuz, Switzerland) to measure the RNA concentration with TaqMan Fast Advanced Master mix together with a Taqman probe specific for the housekeeping gene GUSB (both Thermo Fisher Scientific). A target gene standard curve was generated by creating a fourfold dilution series (range of 0.05–50 ng/µL) of a commercially available standard (HL-60, 100 ng/µL total RNA). The RNA concentrations were calculated by comparing the mean Cq of triplicates measured for test samples to the Cq measured for the different HL-60 dilutions of the standard curve. 

### 4.5. RNA Reverse Transcription

The Superscript Vilo cDNA synthesis kit (Thermo Fisher Scientific) was used for transcription of 10 ng total RNA, as calculated by functional RNA quantification. 

### 4.6. Next Generation Sequencing (NGS)

The Oncomine™ Immune Response Research Assay (Thermo Fisher Scientific) targets and quantifies the expression levels of a panel of 398 immune related genes, including 10 housekeeping genes, using, Ion Torrent (Thermo Fisher Scientific) next generation sequencing (NGS). 

Automated library preparation of 32 samples, each containing 10 ng/µL cDNA, were performed in batches of 4 libraries of 8 samples, according to the manufacturer’s protocol from on the Ion Chef System. The library concentrations were measured by use of the Ion Library TaQMaN quantitation kit (Thermo Fisher Scientific) and the Ion OneTouch™ 2 System was used to prepare the enriched, template-positive Ion PI™ Ion Sphere™ Particles (ISPs). 

In total 10 µL, diluted 1/6 with Tris Low EDTA (Low TE) buffer, from each library were combined. The combined libraries were further diluted to 100 pM and used as a template in the emulsion PCR reaction on the Ion OneTouch™ 2 Instrument, using the Ion PI™ Hi-Q™ OT2 200 kit (Thermo Fisher Scientific). 

Quality control of template-positive Ion spheres (ISPs) was performed on a Qubit™ 2.0 Fluorometer, using the Ion Sphere™ Quality Control Kit, before enrichment of ISPs was performed, using the Ion OneTouch™ ES instrument. 

Target sequencing was performed on an Ion Proton instrument using the Ion PI™ Hi-Q™ Sequencing 200 chemistry and an Ion PI ™ chip (ThermoFisher Scientific). Subsequently, the sequencing results were downloaded to the Affymetrix Transcriptome Analysis Console (TAC) (Thermo Fisher Scientific) for further data analysis of the 27 samples used in the current study. 

### 4.7. Transcriptomic Analysis

Mean housekeeping gene scaled log2 count data from the 398 genes in the Oncomine Immune Response panel, was obtained from the Torrent Suite™ Software (Thermo Fisher Scientific). TAC 4.0.2 was used to analyze differential expression between the two experimental groups. An exploratory grouping analysis provides gene level analysis from small starting material and the workflow involves a two-step process. The first step generates clusters from the data followed by expression analysis to generate fold changes, *p*-values and false discovery rate (FDR) adjusted *p*-values. 

TAC is based on Analysis of variance (ANOVA) and fits a linear model to each probe set independently of the others. We applied a One-Way ANOVA comparison and since the number of samples being analyzed is small, the eBayes analysis corrects the variance of the ANOVA analysis with an empirical Bayes approach that uses the information from all the probe sets to yield an improved estimate for the variance. 

Unsupervised hierarchical clustering of the 27 differentially expressed genes between normal and CIN3 lesions was performed within the ClustVis web tool (https://biit.cs.ut.ee/clustvis/ (accessed on 26 July 2021). The heatmap was generated by applying correlation distance measures and average linkage. 

Gene Set Enrichment Analyses (GSEA) were performed within the JExpress software (www.molmine.com (accessed on 21 October 2020) [51] comparing gene expression patterns in normal versus CIN3/AIS lesions within the C5 (Gene Ontology) gene set collections (Molecular Signature database v4.0 (MSigDB), Broad Institute, Cambridge, MA, USA) [52]. The scoring method for GSEA was Golub (signal-to-noise) permuted on genes.

The IPA analysis revealed relationships between upstream regulators and differentially expressed genes in the dataset by using prediction of activation or inhibition by z- score, ≥2 (activation), and ≤−2 (inhibition).

The PCA was generated with variance scaling applied to rows. Singular value decomposition with imputation was used to calculate the principal components. The ClustVis program was used for visualizing the PCA, illustrating variability in gene expression of the 27 DEGs according to the conditions normal or CIN3/AIS, and also normal or CIN3/AIS according to HPV genotypes.

### 4.8. Relative Quantitative Real Time PCR (qPCR)

qPCR was used to validate the gene expression of CDKN2A and NCAM1 from the NGS analysis. The qPCR was performed on the LightCycler^®^ 480 System with cDNA from 14 normal (Group l) and 13 CIN3/AIS (Group 2). Validation of CDKN2A was performed on cDNA synthesized for the NGS analysis, while new cDNA was synthesized for validation of NCAM1. 

The qPCR was assessed using TaqMan Fast Advanced Master Mix (Life Technologies) and TaqMan probes targeting CDKN2A (hs00923894) and NCAM1 (hs00941830). Based on results from the NGS analysis, the housekeeping gene ABCF1 (hs01073518) was chosen as the internal reference. 

### 4.9. Immunohistochemistry of NCAM1

Optimization of antigen retrieval and dilution of antibody for CD56 was performed prior to the IHC analysis. For all samples, 2 µm thick paraffin sections, adjacent to an HE stained section, were mounted on Superfrost Plus slides (Menzel, Braunschweig, Germany) and incubated for one hour at 60 °C, before being placed in the Dako Omnis autostainer (DAKO Agilent, Santa Clara, CA, USA). After deparaffinization and rehydration, antigen retrieval was performed by use of EnVision Flex (EnV Flex) high PH Tris buffer (DAKO Agilent) at 97 °C for 30 min. The slides were incubated for 20 min with the primary antibody CD56, diluted 1:50 (rabbit monoclonal anti-human MRQ-42; Cell Marque, Rocklin, CA, USA), and followed by Peroxidase treatment in 3 min. 

The immune complex was further visualized by incubation with EnV Flex+ rabbit linker for 10 min., EnV Flex horseradish peroxidase (HRP) for 20 min, and finally incubated in EnV Flex substrate for 5 min. Sections were counterstained with hematoxylin, followed by dehydration in graded ethanol and finally mounted manually. 

The number of positive cells in the epithelium, stroma or both, was assessed by counting the number of positive cells in two neighboring fields of vision (40 × objective 0.52 mm, numerical aperture 0.65) ≈ 1.0 mm^2^. In CIN3 biopsies the most severely dysplastic area with the most intensive p16 staining was interpreted. In normal biopsies an area containing clusters of NCAM1 positive cells was interpreted. The extent and degree of immunopositivity were assessed by consensus scoring by two observers using the same microscope.

### 4.10. Statistics

All probability values were two-sided and considered statistically significant if <0.05. For categorical variables, the correlation between groups was assessed using Pearson χ^2^ or the Fishers exact test as appropriate. For continuous variables, the Mann-Whitney U test and the Independent-Samples T test were applied.

Mean housekeeping gene scaled log2 count data from the 398 genes in the Oncomine Immune Response panel, was obtained from the Torrent Suite™ Software (Thermo Fisher Scientific). TAC 4.0.2 (Applied Biosystem) was used to analyze differential expression between the two experimental groups. An exploratory grouping analysis provides gene level analysis from small starting material and the workflow involves a two-step process. The first step generates clusters from the data followed by expression analysis to generate fold change, *p*-values and FDR adjusted *p*-values. 

The correlation between the mean housekeeping gene scaled log2 NGS data and relative gene expression for CDKN2A and NCAM1 (also designated as CD56 in NCBI Gene) by qPCR, was investigated using the Spearman’s rho correlation coefficient. An Independent-Samples T test was conducted to compare the number of positive NCAM1 cells/1.0 mm^2^ as counted in CIN3/AIS and normal biopsies. Fisher’s exact test was performed to test weak or strong expression in NCAM1 positive cells in the epithelium and the stroma of normal and CIN3/AIS biopsies. 

All statistical analyses, if not otherwise indicated, were performed using IBM SPSS statistics, version 26 (SPSS Inc., Chicago, IL, USA).

## Figures and Tables

**Table 1:** Summary of clinicopathological characteristics before and after the index biopsy for women with normal and CIN3/AIS biopsies (n = 27). The number of cases in each group is given followed by a percentage for each group in parenthesis.

	Normal Biopsies n = 14 (%)	CIN3/AIS Biopsies n = 13 (%)	*p*-Value
Cytology results before index biopsy			0.005 ***
^1^ NILM	9 (100)	0 (0)	
^2^ ASC-US	1 (50)	1 (50)	
^3^ LSIL	2 (50)	2 (50)	
^4^ HSIL	1 (13)	7 (87)	
^5^ ASC-H	1 (33)	2 (67)	
HPV result/genotype before index biopsy *			0.045 ***
^6^ HPV16	5 (45)	6 (55)	
HPV18	0 (0)	3 (100)	
non 16/18 hrHPV	9 (82)	2 (18)	
HPV result/genotype in index biopsy			0.056 ***
HPV16	7 (47)	8 (53)	
HPV18	0 (0)	3 (100)	
non 16/18 hrHPV	2 (50)	2 (50)	
HPV negative	5 (100)	0 (0)	
Cone excision			<0.001 ***
Yes	4 (24)	13 (76)	
No	10 (100)	0 (0)	
Histology results of cone excision			0.001 ***
normal	2 (100)	0 (0)	
^7^ CIN1	2 (100)	0 (0)	
CIN3	0 (0)	11 (100)	
^8^ AIS	0 (0)	2 (100)	
Cytology result after index biopsy **			0.039 ***
Inadequate	1 (100)	0 (0)	
NILM	8 (38)	13 (62)	
ASC-US	3 (100)	0 (0)	
HSIL	1 (100)	0 (0)	
^9^ NA	1 (100)	0 (0)	
HPV result/genotype after index biopsy **			0.288 ***
HPV16	1 (50)	1 (50)	
HPV18	0 (-)	0 (-)	
non 16/18 hrHPV	6 (75)	2 (25)	
HPV negative	6 (38)	10 (62)	
NA	1 (100)	0 (0)	
Median age at diagnosis (range)	33 (26–48)	34 (28–51)	0.367 ****
Median HPV Persistency (days) LBC—last HPV test (range)	1225 (474–2199)	351 (38–1136)	<0.001 ****
Median Total Follow-up (days) from first LBC to last cervical sample within the follow-up period (range)	1336 (717–2199)	985 (414–2072)	0.019 ****

* two LBC samples were not HPV-tested, but HPV16 positive in the index biopsy. ** if several cytology/HPV tests are performed, the result on the last sample is reported. *** Fisher Exact test. **** Mann-Whitney U-test. ^1^ Negative for intraepithelial lesions or malignancy (NILM). ^2^ Atypical squamous cells of undetermined significance (ASCUS). ^3^ Low-grade squamous intraepithelial lesion (LSIL). ^4^ High grade squamous intraepithelial lesions (HSIL). ^5^ Atypical squamous cells, cannot exclude a high-grade lesion (ASC-H). ^6^ Human Papilloma Virus (HPV). ^7^ Cervical intraepithelial neoplasia (CIN). ^8^ Adenocarcinoma in situ (AIS). ^9^ Not Applicable (NA).

**Table 2:** Differentially expressed genes (DEGs) with a fold change >|2| and *p* < 0.05 between normal (n = 14) and CIN3/AIS (n = 13) biopsies identified by Oncomine™ Immune Response Research Assay. Fold change, false discovery rate (FDR) adjusted *p*-values; *p*-values and gene function for the twenty-seven DEGs are included.

Gene Name	Fold Change	FDR *p*-Value	*p*-Value	Gene Function
*CDKN2A*	10.7	0.0000	0.0000	Tumor marker
*KRT7*	4.8	0.0002	0.0000	Tumor marker
*KIAA0101*	2.1	0.0080	0.0001	Epithelial-mesenchymal transition
*MELK*	2.7	0.0128	0.0003	Proliferation
*TOP2A*	2.5	0.0226	0.0006	Epithelial-mesenchymal transition
*CCNB2*	2.4	0.0404	0.0013	Proliferation
*BUB1*	2.4	0.0678	0.0029	Proliferation
*CDK1*	2.6	0.0678	0.0029	Proliferation
*MKI67*	2.6	0.1081	0.0052	Proliferation
*TNFRSF18*	2.0	0.1126	0.0059	Lymphocyte Infiltrate (Tregs)
*FOXM1*	2.3	0.1587	0.0108	Proliferation
*TNFRSF4*	2.4	0.1953	0.0143	Lymphocyte Infiltrate (Tregs)
*CDKN3*	2.0	0.2887	0.0363	Proliferation
*BRCA2*	2.0	0.009	0.0001	Tumor marker
*JCHAIN*	2.25	0.2635	0.02970	B-cell marker
*CMKLR1*	3.02	0.2799	0.03310	Dendritic cell macrophage marker
*NCAM1*	−10.4	0.0000	0.0000	NK-cell
*ARG1*	−11.3	0.0128	0.0002	Macrophage marker
*CD160*	−2.3	0.0678	0.0026	Checkpoint pathways
*CX3CR1*	−2.2	0.0832	0.0038	Lymphocyte Infiltrate
*ALOX15B*	−4.0	0.1108	0.0056	Macrophage marker
*IL18*	−2.3	0.2057	0.0171	T-cell regulation
*CXCL11*	−2.5	0.2635	0.0284	T-cell activation
*CCL5*	−2.1	0.3275	0.0478	Lymphocyte Infiltrate
*LEXM*	−3.14	0.13830	0.00840	T-cell differentiation
*HLA-G*	−3.04	0.26350	0.02680	Antigen processing
*HLA-DQA2*	−2.96	0.26350	0.03050	Antigen processing

**Table 3:** Top ranged C5 Gene ontology gene set collection for human from gene set enrichment analysis (GSEA) comparing normal (n = 14) versus CIN3/AIS (n = 13) lesions. ES (enrichment scores), NES (normalized enrichment scores), Nom *p*-value (nominal *p*-value), FDR (false discovery rate) are included.

Rank	Gene Set	Size	ES	NES	Nom *p*-Value	FDR (%)
1	GO—organelle fission	12	0.88	2.27	0	0
2	GO—regulation of mitotic cell cycle	29	0.69	2.26	0	0
3	GO—mitotic cell cycle	36	0.66	2.25	0	0
4	GO—cell cycle process	42	0.62	2.19	0	0.03
5	GO—positive regulation of cell cycle	19	0.73	2.16	0	0.13
6	GO—regulation of cell cycle process	28	0.67	2.14	0	0.15
7	GO—cell cycle phase transition	24	0.68	2.13	0	0.15
8	GO—cell division	16	0.76	2.12	0	0.17
9	GO—chromosome	28	0.63	2.05	0	0.38
10	GO—chromosome organization	24	0.65	2.02	0	0.63
11	GO—cell cycle arrest	17	0.71	2.02	0	0.59
12	GO—cytoskeletal part	22	0.66	1.98	0	0.92
13	GO—regulation of cell cycle	51	0.54	1.97	0	0.93
14	GO—microtubule cytoskeleton	14	0.74	1.96	0	1.02
15	GO—cell cycle	56	0.52	1.95	0	1.11
16	GO—negative regulation of mitotic cell cycle	17	0.69	1.94	0	1.21
17	GO—negative regulation of DNA binding transcription factor activity	14	0.69	1.91	0	1.68
18	GO—microtubule organizing center	10	0.76	1.87	0	2.74
19	GO—negative regulation of transferase activity	12	0.71	1.86	0.01	2.89
20	GO—negative regulation of cell cycle process	13	0.69	1.85	0	2.97
21	GO—negative regulation of cell cycle phase transition	10	0.75	1.82	0	3.93
